# Improving Physical Task Performance with Counterfactual and Prefactual Thinking

**DOI:** 10.1371/journal.pone.0168181

**Published:** 2016-12-12

**Authors:** Cecilia Hammell, Amy Y. C. Chan

**Affiliations:** School of Psychology, University of Wollongong, North Wollongong, New South Wales, Australia; Waseda University, JAPAN

## Abstract

Counterfactual thinking (reflecting on “what might have been”) has been shown to enhance future performance by translating information about past mistakes into plans for future action. Prefactual thinking (imagining “what might be if…”) may serve a greater preparative function than counterfactual thinking as it is future-orientated and focuses on more controllable features, thus providing a practical script to prime future behaviour. However, whether or not this difference in hypothetical thought content may translate into a difference in actual task performance has been largely unexamined. In Experiment 1 (*n* = 42), participants performed trials of a computer-simulated physical task, in between which they engaged in either task-related hypothetical thinking (counterfactual or prefactual) or an unrelated filler task (control). As hypothesised, prefactuals contained more controllable features than counterfactuals. Moreover, participants who engaged in either form of hypothetical thinking improved significantly in task performance over trials compared to participants in the control group. The difference in thought content between counterfactuals and prefactuals, however, did not yield a significant difference in performance improvement. Experiment 2 (*n* = 42) replicated these findings in a dynamic balance task environment. Together, these findings provide further evidence for the preparatory function of counterfactuals, and demonstrate that prefactuals share this same functional characteristic.

## Introduction

People often imagine how an outcome of a past event might have been different (counterfactual thinking) and how things might be different in the future (prefactual thinking). Commonly elicited by a negative event (e.g., losing in a race), counterfactual and prefactual thoughts take the form of “if-then” conditional propositions in which the “if” specifies an alternative action (e.g., “if only I had trained harder” for counterfactuals; “if only next time I train harder” for prefactuals) and the “then” specifies an imagined outcome or goal (e.g., “then I would have won/then I will win the race”). Accordingly, such thoughts allow one to consider different courses of action that may have led, or may lead, to a more desirable outcome, preparing them to implement such actions if a comparable situation presents itself in the future [1].

Epstude and Roese [1] proposed a functional theory of counterfactual thinking. This theory postulates that counterfactuals may enhance performance by way of either a content-neutral pathway that facilitates a broadened mindset or heightened motivation to consider multiple alternatives, or more directly via a content-specific-pathway that identifies specific alternative, more adaptive courses of action which, in turn, lead to a shift in behavioural intentions. The functional value of counterfactual thinking via this content-specific pathway is well supported. Context-related counterfactual thoughts have been shown to activate behavioural intentions [2–4] and subsequently facilitate behaviour change and performance improvement. This has been demonstrated in a variety of contexts including anagram task performance [3], exam performance [5], and simulated aircraft landing [6]. Several factors, however, have been shown to affect the functionality of counterfactuals. For example, additive (imagining a new action) and upward (imagining how things could have turned out better) counterfactuals increase the likelihood of improved future outcomes relative to either subtractive (omitting a previous action) or downward (imagining how things could have been worse) counterfactuals [3]. More recently, the content of counterfactual thoughts has been shown to influence whether or not the counterfactual will elicit the corresponding behavioural intention. Specifically, counterfactual thoughts following a negative event (e.g., bad sunburn) that contain more detailed information and involve specific behaviours (e.g., practicing good skin care) rather than personal qualities (e.g., being more responsible) are more likely to translate into behavioural intentions (e.g., wearing sunscreen in the future) [7].

While in some instances reflection on past events may occur in the process of generating simulations of future events, research has shown that people are capable of generating ‘what if’ thoughts to anticipate what may happen in the future without having had any prior experience [8, 9]. Thus, counterfactual and prefactual thinking appear to be two distinct thought processes. Under the umbrella construct of prospection, a range of future-oriented cognitions have been examined in relation to future behaviour. For example, episodic future simulation and affective forecasting in decision making [10–13], and implementation intentions and performance task improvement [14, 15]. While these processes all involve the hypothetical construction of an imagined future event, Epstude, Scholl and Roese [16] argue that prefactual thinking is a distinct mode of prospection. Specifically, rather than purely simulating or predicting the future, a key distinction is that prefactuals provide a conditional (if-then) proposition of a future action-outcome link. Implementation intentions, although similar in that they also specify future-outcome linkages, identify a particular circumstance (e.g., “when I get home this afternoon”) in which a particular action (e.g., “I will go for a run”) will be carried out to achieve a goal (e.g., “to become fitter”). Prefactual thoughts, on the other hand, offer more general action-outcome linkages that may (or may not) occur in the future (e.g., “If I go for a run then I will become fitter”). In this sense, prefactual thinking may offer a more flexible, generalisable and enduring prescription for future behaviour. Byrne [17] identified a relative lack of research on prefactual thinking and raised the question of whether prefactuals share the same characteristics as counterfactuals. While the majority of research on prefactual thinking has been concerned with anticipated regret and decision-making [18, 19], an interest in the possible functions of prefactuals compared with counterfactuals has recently emerged. Most notably, Ferrante, Girotto, Stragà and Walsh [20] examined the content of both counterfactual and prefactual thoughts following task failure, and found that counterfactuals more often focused on undoing features that were beyond immediate control (e.g., situational factors, personality traits, or psychophysical status). Prefactual thoughts, on the other hand, concentrated more on controllable elements (e.g., concentration level or specific strategies to improve skills). It was reasoned that in imagining how things might be different in the future, individuals become more aware that the constraints that governed their past attempt (task demands, available resources, and their own skills) will also be present in the future. Given that these features cannot be changed in reality, they are not considered as viable candidates for undoing in prefactual mental simulations and thus only controllable features are imagined. Stragà and Ferrante [21] replicated this temporal asymmetry in hypothetical thought content among athletes who had just completed a marathon, and further found that a focus on training was associated with a greater intention to train harder for future marathons among those who generated prefactual thoughts, but not for those who generated counterfactuals. These results raised questions regarding the relative utility of counterfactuals and prefactuals in supporting improved future behaviour.

While this research has increased our understanding of the *content* of counterfactual and prefactual thoughts and how this may influence the formation of behavioural plans, the downstream effects of this on actual behaviour is unknown. Akin to the way that implementation intention plans have been shown to facilitate translation of intention to behaviour and enhance subsequent behaviour modification [see 14, 15], we reasoned that as prefactual thinking is future-oriented and focuses on more controllable features, it should provide a more precise roadmap for future behaviour, and hence, via the content-specific pathway, prime and trigger future behaviour more directly should a similar situation arise in the future. We predicted that forming task-related prefactual thoughts should serve a greater functional benefit than counterfactual thinking for future task performance.

The present study aimed to replicate previous findings regarding the content of counterfactual and prefactual thoughts. Additionally, we sought to investigate whether or not this difference in thought content may further translate into a difference in actual task performance. In line with the findings of Ferrante et al. [20] and Stragà and Ferrante [21], it was hypothesised that the content of prefactual thoughts would focus on more controllable aspects of a task compared with that of counterfactual thoughts. Moreover, it was hypothesised that both counterfactual and prefactual thinking would result in improvement in subsequent task performance, however, this improvement would be more pronounced for those who generated prefactual thoughts. These hypotheses were tested in two experiments. In Experiment 1, participants performed trials of a computer-simulated physical task on the Nintendo Wii™ console, in between which they engaged in either task-related hypothetical thinking (counterfactual or prefactual) or an unrelated filler task (control). Experiment 2 aimed to replicate the findings from Experiment 1 in a dynamic balance task environment in which participants performed balance trials on a wobble board.

## Experiment 1

### Methods

#### Participants

Forty-two undergraduate psychology students from the University of Wollongong aged between 17 and 43 years (*M* = 21.83 years, *SD* = 6.12 years, 29 females, 13 males) volunteered to participate in exchange for partial course credit. Prior to involvement, all participants provided written informed consent. As per participants’ self-reports, all participants had minimal experience playing Wii-Sport or games that require shooting at a target on the Wii console, and were novices in the sport of archery. Participants were randomly assigned to one of three thought conditions (*n* = 14 in each condition): counterfactual thinking (CFT; 5 females), prefactual thinking (PFT; 9 females) or a control condition (9 females).

Two participants were aged 17 years at the time of their participation. In considering our research, the University of Wollongong Human Research Ethics Committee (HREC) referred to section 4.2 of the National Statement on Ethical Conduct in Human Research which outlines the circumstances under which people under the age of 18 can give consent for their own participation in research (e.g., if “he or she is mature enough to understand the relevant information and give consent”, “the research involves no more than low risk”). It is on this basis that the HREC approved the participation of participants under the age of 18 and the written consent procedure aforementioned that was followed in the current research (research protocol HE14/041).

#### Computer-simulated physical task

Performance was assessed using a computer-simulated game of archery provided by Wii-Sports (Resort) and played on the Nintendo-Wii™ console. In each game trial, participants completed the intermediate level of the game, which consisted of four stages of increasing difficulty (marked by changes in target distance, wind speed, and wind direction), with three arrows to shoot per stage. Participants stood 2 m away from the television game screen (48 x 17 cm). To shoot, they had to mimic the necessary motor movements required to perform the task in real life (drawing the arrow and aiming), which were monitored by a motion sensor bar above the screen. The game prompted which buttons to press during the actions and indicated on the screen the distance of the target (ranging from 15 to 40 m), which increased over stages, wind speed (ranging from 0 to 6 mph), and wind direction (left, right, or upward), which changed randomly over the stages. The target consisted of 10 evenly spaced concentric rings with scores ranging from 10 for the bullseye, to 1 for the outermost ring. Missing the target resulted in 0 points. The value of each target ring hit in each stage was totalled to provide the overall performance score for that trial.

#### Condition tasks

To prompt hypothetical thinking following performance trials, participants were given a worksheet that instructed them to write down as many thoughts as possible in 2 ½ minutes to complete the sentence, ‘My score in the game *would* have been better if…’ for the CFT condition or ‘My score in that game *will* be better next time if…’ for the PFT condition. For participants in the control condition, two activities in the figural subtest of the Torrance Test of Creativity (the ‘picture completion’ activity and the ‘lines’ activity) [22] were used as a cognitively engaging filler task. Each activity in this task required participants to add lines to several incomplete figures or lines to create a unique picture, after which they had to create an interesting title for each drawing. This task was administered to participants in the control condition as a substitute for the hypothetical thought task, and was intended to minimise the possibility of control participants spontaneously generating any hypothetical thoughts.

#### Baseline motivation scale

Participants’ baseline motivation was measured with a single item (“Please use the scale below to indicate how motivated you are to perform well on this game”) using a 5-point rating scale ranging from 1 (*not at all motivated*) to 5 (*very motivated*).

### Procedure

Participants were tested individually over a single session taking approximately 30 minutes. After providing written consent, participants were shown the start game screen and the game was explained (see S1 Instructions). A practice trial was then performed which repeated the verbal instructions given and allowed participants to become familiar with how to play the game. After this, participants provided their brief demographic information, completed the baseline motivation scale and were directed back to the game for the first game trial.

Participants were notified of their score following the game trial. The task intended to prompt hypothetical thoughts specific to participants’ condition was then administered to those in the CFT and PFT conditions. Participants were instructed to complete this task in reference to the game trial they had just completed. After completing the written task, participants were redirected back to the game and performed the next game trial. This procedure was repeated once more so that all participants completed two trials of their condition task and three game trials.

Participants in the control condition were instructed to take a break before their next turn of the game. As a cover story for the unrelated control group task, the experimenter explained that they needed to calibrate how much time to allow people to complete a drawing task for an unrelated experiment. The experimenter asked participants if, whilst on their break between game trials, they could try to complete the ‘picture completion’ drawing activity in 2 ½ minutes. All participants agreed to complete this activity. The drawing activity was then administered. Afterwards, participants were redirected back to the game and performed the second game trial. Following the second game trial the ‘lines’ drawing activity was administered, after which the third game trial was performed. All participants were then debriefed, thanked, and dismissed.

### Results and Discussion

#### Coding hypothetical thoughts

Based on Tsiro and Mittal’s [23] criteria for classifying counterfactuals, responses that “alter reality, create hypothetical scenarios, or express an opinion as to what might have been had a different decision been made” were counted as counterfactual thoughts. Responses of the same nature that nonetheless expressed an opinion as to what might be if a different decision were to be made in the future were classified as prefactual thoughts. The responses were further coded according to Ferrante et al.’s [20] criteria for classifying thoughts as controllable, uncontrollable, or other (see S1 Table for criteria and examples). Participants in the CFT and PFT conditions generated a total of 191 thoughts. Of these thoughts, 5 were coded as ‘other’ and were thus excluded, leaving a total of 186 hypothetical thoughts. Two independent raters coded the responses according to these criteria. Inter-rater agreement was 96%, Cohen’s κ = .95, *p* < .001. Disagreements were resolved through discussion. The results are shown in Table 1.

**Table 1:** Mean Age, Practice Trial Performance, Baseline Motivation and Counterfactual and Prefactual Controllable and Uncontrollable Thoughts Generated by the CFT and PFT Condition in Experiment 1.

	CFT			PFT			Control			
Variable	M	(*SD*)	95% CI	M	(*SD*)	95% CI	M	(*SD*)	95% CI	*F* (df)
**Age**	19.29	(1.82)	[16.06, 22.51]	22.50	(6.79)	[19.27, 25.73]	23.71	(7.59)	[20.49, 26.94]	5.17 (2, 39)
**Practice Trial Performance**	18.07	(6.26)	[14.44, 21.71]	17.79	(6.60)	[14.15, 21.42]	16.43	(7.28)	[12.80, 20.07]	0.18 (2, 39)
**Baseline Motivation**	3.86	(.77)	[3.53, 4.19]	4.07	(.47)	[3.74, 4.40]	4.14	(.53)	[3.82, 4.47]	1.05 (2, 39)
**Thoughts Generated**										
** CFT Controllable**	4.21	(2.64)	[2.69, 5.74]	0.21	(0.43)	[-0.03, 0.46]				
** CFT Uncontrollable**	2.71	(1.82)	[1.67, 3.76]	0.14	(0.36)	[-0.07, 0.35]				
** PFT Controllable**	0.00	(0.00)	[0.00, 0.00]	5.21	(2.55)	[3.74, 6.69]				
** PFT Uncontrollable**	0.00	(0.00)	[0.00, 0.00]	0.79	(1.37)	[-0.01, 1.58]				

Note. CFT = Counterfactual thinking; PFT = Prefactual thinking

#### Content of thoughts

Participants generated thoughts appropriate to their respective experimental condition: those in the CFT condition only generated counterfactual thoughts and those in the PFT condition generated predominantly prefactual thoughts (see Table 1). To examine the content of the thoughts, two separate (controllable x uncontrollable thoughts) paired sample t-tests were conducted for counterfactuals generated within the CFT condition and for the prefactuals generated within the PFT condition respectively. On average, participants in the CFT condition were equally as likely to generate thoughts about uncontrollable (*M* = 4.00, *SD* = 2.32) and controllable (*M* = 1.79, *SD* = 2.52) task elements, *t*(13) = 1.92, *p* > .05, *η*^2^ = .13. However, in support of Ferrante et al. [20] and our first hypothesis, participants in the PFT condition were more likely to generate thoughts about controllable (*M* = 6.71, *SD* = 2.79) rather than uncontrollable (*M* = .21, *SD* = .58) task elements, *t*(13) = 8.79, *p* < .001, *η*^2^ = .40.

#### Hypothetical thinking and task performance

**Fig 1:**
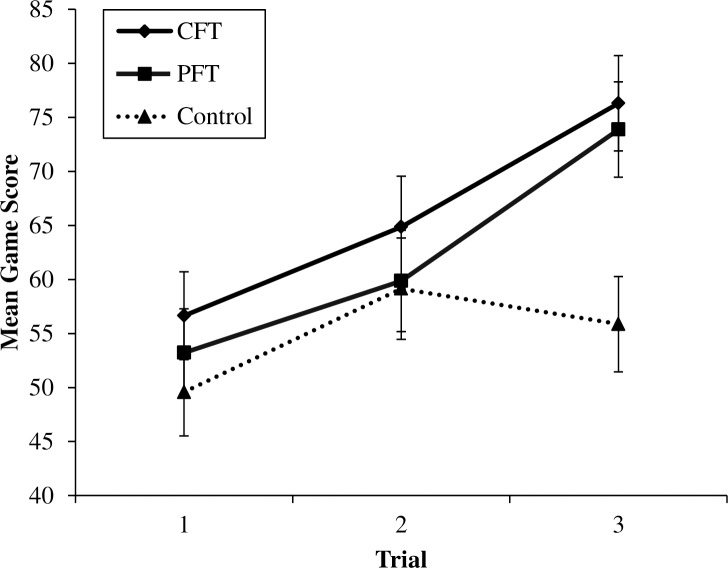
Mean game scores across trials for the counterfactual (CFT), prefactual (PFT), and control condition in Experiment 1. Error bars indicate standard errors.

A series of separate univariate Analysis of Variance (ANOVA) indicated no systematic differences across experimental conditions in participants’ sex, practice performance, or baseline motivation (see Table 1 for descriptive information). Age was found to vary across conditions, however, a subsequent Analysis of Covariance (ANCOVA) did not show age to be a significant covariate and thus, task performance was analyzed using a 3 (thought condition) x 3 (performance trial: 1, 2, 3) mixed factorial ANOVA. A Bonferroni-adjustment was applied to all follow-up pairwise comparisons to control the overall alpha level at .05. The results revealed a main effect of trial, *F*(2, 78) = 43.10, *p* < .001, *partial η*^2^ = .53, but not of thought condition, *F*(2, 39) = 1.93, *p* > .05, *partial η*^2^ = .09. There was, however, a significant interaction between trial and thought condition, *F*(4, 78) = 6.09, *p* < .001, *partial η*^2^ = .24. Both the CFT and PFT conditions improved significantly in performance across successive trials (i.e., 1 and 2, 2 and 3 and 1 and 3) (*p* < .05 for all pairwise comparisons) whereas the control condition improved across trial 1 and 2 (*p* < .05) but not across trials 2 and 3 (*p* = .84) and 1 and 3 (*p* = .16). These results are displayed in Fig 1.

These findings extend upon previous work [20, 21] and contribute to the existing literature on the functions of counterfactual literature by providing preliminary support for a shared functional benefit of prefactual thinking. Additional research is warranted to determine if the present findings can be generalised from the context of a simulated physical task to an actual physical task setting.

The full dataset is available in the S2 Table file. Any personal information has been removed and only raw scores are provided.

## Experiment 2

### Methods

#### Participants

Forty-two participants aged between 18 and 40 years (*M* = 22.98 years, *SD* = 4.80 years, 30 females, 12 males) took part. Participants were recruited from the University of Wollongong and general community either via an online research system as a means of achieving course credit (*n* = 9), via advertisements around the university campus (*n* = 25), or were students known to the experimenter but were nonetheless unaware of the details of the experiment (*n* = 8). Written informed consent was obtained from all participants prior to involvement. All participants were volunteers, and reported that they had minimal experience using a wobble-board, were free from injuries that may have compromised their balance ability, and had not participated in Experiment 1. As in Experiment 1, participants were randomly assigned to the three experimental conditions (CFT, PFT, or control; 10 females and 4 males in each condition). All participants were above the age of 18. This study and the consent procedure aforementioned were approved by the University of Wollongong Human Research Ethics Committee (research protocol HE14/289).

#### Materials and task

The performance task required participants to balance on a 66fit® wobble-board. The apparatus consisted of a flat circular surface (46 cm in diameter) that stood 9 cm off the ground, with a maximum tilt angle of 18 degrees to the left and right sides. A dark piece of tape (20 cm x 1 cm) was positioned horizontally on the wall (1.5 m from the ground), which served as an external focus point for participants to look at whilst balancing. Balance time (i.e., surface angle was not in contact with the ground) was measured to the nearest 1/100^th^ of a second. Baseline motivation was measured using the same scale used in Experiment 1. The task used to prompt counterfactual and prefactual thinking was also the same as in Experiment 1, however, was reworded to accommodate the change of task: ‘My performance time in that balance task *would* have been better if…’ for the CFT condition or ‘My performance time in that balance task *will* be better next time if…’ for the PFT condition. As per Experiment 1, the figural subtest of the Torrance Test of Creativity [22] was used as a filler task in between balance trials for participants in the control condition.

### Procedure

Participants were tested individually over a single session taking approximately 25 minutes. Following written consent, participants were directed to the wobble-board for a practice trial. Participants were instructed to perform the task barefoot, place their feet apart on the wobble-board surface and “look straight ahead and focus on keeping the wobble-board surface horizontal, in line with the tape on the wall”. To increase participants’ motivation to perform well on the task, they were informed that “participants who achieve the top 10 best times will be entered into a draw to win the wobble-board at the end of the study”. The practice trial consisted of five attempts (as did each of the three test trials that followed). The experimenter signalled the onset of each attempt and encouraged participants to take their time when starting. The timer was started when the board left the ground and stopped when it made contact with the ground. The median balance time from the attempts in each trial was used as the overall performance time for that trial.

Following the practice trial, participants’ demographic information was collected and they completed the baseline motivation scale. Participants were then asked to perform the first balance trial. Upon completion participants were notified of their best balance time. The task specific to participants’ experimental condition was then administered as per Experiment 1. Participants were redirected back to the balance task and performed their next trial. The procedure, as specified above, was repeated once more, until two trials of the thought condition task and three balance trials were completed. Participants were debriefed, given their token of thanks (either a coffee voucher or course credit), and dismissed.

### Results and Discussion

#### Content of thoughts

Participants in the CFT and PFT conditions generated a total of 198 thoughts. Of these thoughts, 2 were coded as ‘other’ and were thus excluded, leaving a total of 196 hypothetical thoughts. Inter-rater agreement of thought responses was 90%, Cohen’s κ = .85, *p* < .001. Discrepancies were resolved through discussion. Thought responses (see Table 2) were examined using two separate 2 (type of hypothetical) x 2 (controllability) repeated measures ANOVA for the CFT condition and the PFT condition respectively. As expected, there was a significant main effect of type of hypothetical thoughts generated for both the CFT condition, *F*(1, 13) = 47.59, *p* < .001, *partial η*^2^ = .76, and the PFT condition, *F*(1, 13) = 23.54, *p* < .001, *partial η*^2^ = .64. Bonferroni-adjusted pairwise comparisons confirmed that responses generated by the CFT condition were predominantly counterfactual (*p* < .001), and predominantly prefactual for the PFT condition (*p* < .001). A significant main effect was also found for controllability for the PFT condition, *F*(1, 13) = 91.32, *p* < .001, *partial η*^2^ = .88, but not for the CFT condition *F*(1, 13) = 3.15, *p* = .10, *partial η*^2^ = .20. In line with Experiment 1 and in further support of Ferrante et al.’s [20] findings, the PFT condition generated significantly more controllable thoughts compared to uncontrollable thoughts (*p* < .001), whereas the CFT condition did not (*p* = .08). These results are displayed in Table 2.

**Table 2:** Mean Age, Practice Trial Performance, Baseline Motivation and Counterfactual and Prefactual Controllable and Uncontrollable Thoughts Generated by the CFT and PFT Condition in Experiment 2.

	CFT			PFT			Control			
Variable	M	(*SD*)	95% CI	M	(*SD*)	95% CI	M	(*SD*)	95% CI	*F* (df)
**Age**	23.57	(4.42)	[20.93, 26.22]	22.86	(4.70)	[20.21, 25.50]	22.50	(5.49)	[19.86, 25.14]	0.01 (2, 39)
**Practice Trial Performance**	10.04	(9.19)	[5.51, 14.58]	9.25	(11.06)	[4.72, 13.79]	5.28	(2.15)	[0.75, 9.82]	3.54 (2, 39)
**Baseline Motivation**	4.07	(0.27)	[3.72, 4.43]	3.93	(0.92)	[3.57, 4.28]	3.93	(0.62)	[3.57, 4.28]	0.86 (2, 39)
**Thoughts Generated**										
** CFT Controllable**	4.00	(2.32)	[2.66, 5.34]	0.57	(1.65)	[-0.38, 1.53]				
** CFT Uncontrollable**	1.77	(2.52)	[0.33, 3.24]	0.21	(0.80)	[-0.30, 0.68]				
** PFT Controllable**	0.21	(0.58)	[-.012, 0.55]	6.71	(2.78)	[5.11, 8.32]				
** PFT Uncontrollable**	0.29	(1.07)	[-0.33, 0.90]	0.21	(0.58)	[-0.12, 0.55]				

Note. CFT = Counterfactual thinking; PFT = Prefactual thinking

#### Hypothetical thinking and task performance

A series of separate univariate ANOVAs indicated no systematic differences across experimental conditions in participants’ age, sex, or practice trial performance. Baseline motivation, however, did vary across conditions (see Table 2 for descriptive information). Given that a subsequent ANCOVA did not show baseline motivation to be a significant covariate, the main hypotheses were examined using a 3 (thought condition) x 3 (performance trial: 1, 2, 3) mixed factorial ANOVA. A Bonferroni-adjustment was applied to all follow-up pairwise comparisons to control the overall alpha level at .05. The Greenhouse-Geisser correction is used as the assumption of homogeneity of variance was not met.

The results revealed a significant main effect of trial, *F*(1.77, 69.14) = 44.50, *p* < .001, *partial η*^2^ = .53, and of thought condition, *F*(2, 39) = 4.41, *p* < .05, *partial η*^2^ = .18. Averaged across trials, there was no significant difference in participants’ task performance between the CFT and PFT conditions (*M*_*Diff*_ = 2.06, *p* > .05). The control condition, however, performed significantly poorer than the CFT condition (*M*_*Diff*_ = -5.52, *p* < .05) and the PFT condition (*M*_*Diff*_ = -3.46, *p* < .05).

**Fig 2:**
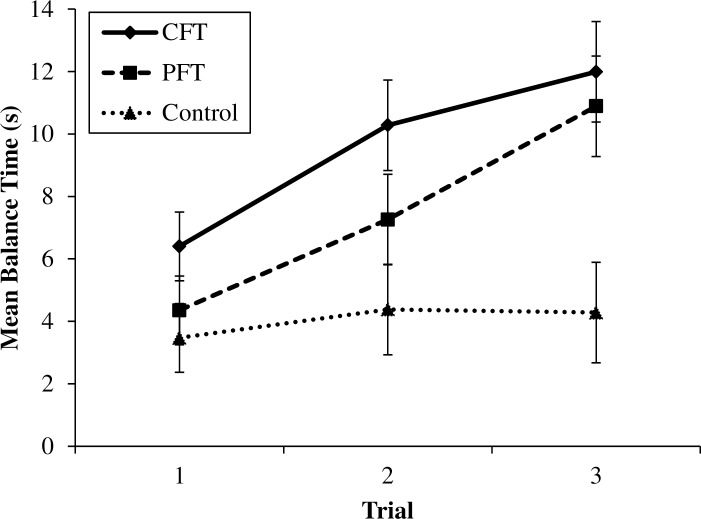
Mean balance times across trials for the counterfactual (CFT), prefactual (PFT), and control condition in Experiment 2. Error bars indicate standard errors.

Additionally, there was a significant interaction between trial and thought condition, *F*(3.55, 69.14) = 8.00, *p* < .001, *partial η*^2^ = .29. In line with Experiment 1, both the CFT and PFT conditions improved significantly in performance across successive trials (i.e., 1 and 2, 2 and 3 and 1 and 3) (*p* < .05 for all pairwise comparisons) whereas the control condition did not improve across any of the trials (*p* ≥ .95 for all pairwise comparisons). These results are displayed in Fig 2.

The full dataset is available in the S3 Table file. Any personal information has been removed and only raw scores are provided.

## General Discussion

This study examined the preparative functions of prefactual thinking compared with counterfactual thinking. In line with previous findings [20, 21], we found that for participants who generated task-related prefactual thoughts, there was a greater focus on mutating controllable task elements. Those who generated counterfactual thoughts were also likely to mutate controllable task elements, although they were equally likely to generate thoughts about uncontrollable task elements. We further extended on previous research by investigating whether or not this difference in thought content would translate into a difference in actual task performance. Contrary to the doubt raised regarding the relative preparatory values of counterfactual and prefactual thoughts, we have demonstrated in two experiments that PFT and CFT are similarly effective in facilitating improvement in task performance.

Counter to our expectation, prefactual thinking did not yield more pronounced task improvement than counterfactual thinking. Perhaps the tendency for participants in both hypothetical thought conditions to generate thoughts on controllable features may have enabled prefactual and counterfactual thinking to be similarly effective. Alternatively, it is possible that the hypothetical thoughts that did not provide specific or controllable information still influenced future behaviour via more general processing mechanisms (i.e., the content-neutral pathway) such as through motivation and mindset (rather than via a shift in behavioural intentions; i.e., the content-specific pathway). Further research is warranted to measure participants’ intention to alter their performance behaviour according to the thoughts they had generated *as well as* their actual task performance. This would provide deeper insight into the processes by which counterfactuals and prefactuals influence behaviour and would allow for a more accurate comparison of the preparative function of prefactuals and counterfactuals specifically by way of the content-specific pathway.

More broadly, future research is encouraged to consider the ways in which counterfactual and prefactual thinking may differ in the way they interact with other facets of learning and goal-directed behaviour change. Wulf, Chiviacowsky and Lewthwaite [24] demonstrated that performance and learning of a balance task among older adults could be influenced positively, and almost immediately, by enhancing individuals’ self-efficacy with a simple statement suggesting that their peers generally perform well on the task. Counterfactual and prefactual thinking may be distinct in the way that they operate together with other goal cognitions such as motivation and self-efficacy. In particular, a greater conceptual overlap between prefactual thinking and self-efficacy (i.e., they are both future-orientated) may be reason to speculate a combination of prefactual thinking and higher self-efficacy would have greater adaptive benefits for future performance compared with a combination of counterfactual thinking and self-efficacy.

It is important to acknowledge that we assessed the functions of prefactual and counterfactual thinking in task environments that may not generalise to more ‘natural’ learning settings. Previous research has indicated that the preparative function of counterfactual thinking is sensitive to changes in temporal distance, such that it has greater influence when either the negative event occurred in the recent past, or the behavioural intention occurs in the near future [25]. Given that prefactual thoughts are future-orientated, it is possible that the behavioural prescriptions they offer are less sensitive to time. Thus, in the context of predicting adaptive behaviour towards the next marathon, as in the context of Stragà and Ferrante’s [21] study, the relative benefits of prefactual and counterfactual thinking remain unclear. Our experiments provided a more direct test to compare the immediate effects of prefactual and counterfactual thinking on task performance, in the same tradition as Roese’s [3] anagram studies. Future research in this area is encouraged to explore how the preparatory functions of counterfactual and prefactual thinking may differ in contexts in which skill acquisition and performance improvement occur gradually over a period of time (e.g., learning to play a musical instrument, studying for a difficult subject, or training to be a better marathon runner).

In this study, we empirically demonstrated the utility of prefactual thinking in facilitating future performance behaviour and thus can offer a preliminary answer to Byrne’s [17] question: Yes, prefactuals do appear to share a similar functional characteristic to counterfactuals. To our knowledge, this is the first study to examine prefactual compared to counterfactual thinking in light of its functional value for future performance. While our small sample size is a limitation, it is noteworthy that our results are consistent across the two experiments. Both of these experiments have demonstrated that while it can be helpful to learn from reflecting on past experiences and mistakes (i.e., counterfactual thinking), contemplation of potentially successful behaviours (i.e., prefactual thinking) can be equally beneficial to future performance. In light of this, prefactual thinking may have practical implications for learning and performance particularly in circumstances where people may not have the opportunity to learn from previous experience (e.g., performing a task for the first time). Moreover, our findings suggest that both counterfactual and prefactual thinking are useful strategies to enhance balance performance. Further research is warranted to examine counterfactual and prefactual thinking as potential metacognitive interventions to support balance improvement, perhaps among the older adult population.

## Supporting Information

S1 Instructions Game Instructions Experiment 1. (DOCX)

S1 Table Classification Criteria for Coding Counterfactual and Prefactual Thoughts. (DOCX)

S2 Table Dataset Experiment 1. (DOCX)

S3 Table Dataset Experiment 2. (DOCX)
