# Correction: Improving Physical Task Performance with Counterfactual and Prefactual Thinking

**DOI:** 10.1371/journal.pone.0231278

**Published:** 2020-03-27

**Authors:** Cecilia Hammell, Amy Y. C. Chan

Under the Content of thoughts subheading in the Results and Discussion subsection of Experiment 1, there are errors in the third and fourth sentences. The correct sentences are: On average, participants in the CFT condition were equally as likely to generate thoughts about uncontrollable (*M* = 2.71, *SD* = 1.82) and controllable (*M* = 4.21, *SD* = 2.64) task elements, *t*(13) = 1.49, *p* > .05, *d* = .40. However, in support of Ferrante et al. [20] and our first hypothesis, participants in the PFT condition were more likely to generate thoughts about controllable (*M* = 5.21, *SD* = 2.55) rather than uncontrollable (*M* = .79, *SD* = 1.37) task elements, *t*(13) = 5.06, *p* < .001, *d* = 1.35.
