# Supplementary material for: Improving Physical Task Performance with Counterfactual and Prefactual Thinking
Source: PLoS One. 2016 Dec 12;11(12):e0168181. doi: 10.1371/journal.pone.0168181 (PMC5152910; doi:10.1371/journal.pone.0168181)
Supplement: S1 Instructions — (DOCX) [file pone.0168181.s001.docx]

**S1 Instructions. Full Game Instructions Experiment 1.**

*“In this game, the goal is to hit the target as close to the centre as possible. The closer you get, the more points you will receive, with the bull’s eye being worth 10 points. Missing the target will result in 0 points. To play, hold the Wii-remote in your left hand and the Wii-nunchuck in your right hand, or vice versa if you are left handed. Stand side-on to the screen and hold the controllers in front of your body. To shoot an arrow, hold down button A on the remote and button Z on the nunchuck, and pull the nunchuck back as though you are drawing an arrow in real life. A circle will appear which you can use to take aim. To shoot, release the Z button. When lining up your shaft, be aware of the wind direction, wind speed, and target range indicated in the top right corner of the screen, as these change and can alter the path of your arrow. There are 4 stages to each game trial, and you have 3 arrows to shoot per stage. Before we start the real game, I will get you to have a go at the first stage as a practice”.*
