# Supplementary material for: Improving Physical Task Performance with Counterfactual and Prefactual Thinking
Source: PLoS One. 2016 Dec 12;11(12):e0168181. doi: 10.1371/journal.pone.0168181 (PMC5152910; doi:10.1371/journal.pone.0168181)
Supplement: S1 Table — (DOCX) [file pone.0168181.s002.docx]

**S1 Table. Classification Criteria for Coding Counterfactual and Prefactual Thoughts.**

| **Classification** | **Criteria** | **Example*** |
| --- | --- | --- |
| **Controllable** | Concentration and attention: | “If I concentrate/d harder” |
|  | Reasoning strategies and tactics: | “If I aim/ed the bow higher” |
| **Uncontrollable** | Problem features: | “If the target is/was closer” |
|  | Psychophysical status: | “If I am not/was not tired” |
|  | Stable traits: | “If I am/was more coordinated” |
|  | Things that could not be improved before the next game: | “If I have/had more practice” |
|  | The context: | “If I am/was alone” |
| **Other** | Non-informative/ambiguous responses: | “If I take/took this game seriously” |

Note. *Example prefactual thought and corresponding counterfactual thought
