# Supplementary material for: Improving Physical Task Performance with Counterfactual and Prefactual Thinking
Source: PLoS One. 2016 Dec 12;11(12):e0168181. doi: 10.1371/journal.pone.0168181 (PMC5152910; doi:10.1371/journal.pone.0168181)
Supplement: S2 Table — (DOCX) [file pone.0168181.s003.docx]

**S2 Table. Dataset Experiment 1.**

| **Participant** | **Age** | **Sex** | **Glasses** | **Thought Condition** | **Baseline Motivation** | **Practice** | **Trial 1** | **Trial 2** | **Trial 3** | **Thoughts Generated** |
| --- | --- | --- | --- | --- | --- | --- | --- | --- | --- | --- |
| 1 | 20 | 2 | 1 | 3 | 4 | 13 | 40 | 43 | 52 | 0 |
| 2 | 23 | 1 | 2 | 2 | 4 | 14 | 66 | 62 | 82 | 8 |
| 3 | 23 | 2 | 2 | 1 | 5 | 8 | 45 | 43 | 30 | 4 |
| 4 | 23 | 2 | 2 | 2 | 4 | 16 | 52 | 54 | 76 | 8 |
| 5 | 20 | 2 | 2 | 3 | 4 | 17 | 58 | 43 | 45 | 0 |
| 6 | 20 | 1 | 1 | 1 | 2 | 15 | 65 | 59 | 84 | 4 |
| 7 | 26 | 2 | 1 | 3 | 4 | 25 | 71 | 72 | 80 | 0 |
| 8 | 18 | 2 | 2 | 1 | 4 | 20 | 45 | 47 | 57 | 9 |
| 9 | 29 | 2 | 2 | 2 | 4 | 21 | 56 | 87 | 80 | 7 |
| 10 | 23 | 2 | 3 | 2 | 4 | 14 | 41 | 42 | 49 | 5 |
| 11 | 21 | 1 | 2 | 1 | 3 | 15 | 35 | 44 | 78 | 5 |
| 12 | 39 | 2 | 1 | 3 | 4 | 19 | 28 | 32 | 14 | 0 |
| 13 | 32 | 1 | 1 | 3 | 5 | 17 | 53 | 70 | 58 | 0 |
| 14 | 19 | 1 | 2 | 2 | 5 | 27 | 72 | 79 | 92 | 3 |
| 15 | 17 | 2 | 3 | 1 | 4 | 4 | 50 | 55 | 79 | 8 |
| 16 | 19 | 1 | 2 | 3 | 5 | 25 | 94 | 103 | 84 | 0 |
| 17 | 43 | 1 | 1 | 2 | 4 | 7 | 18 | 28 | 64 | 12 |
| 18 | 18 | 2 | 3 | 1 | 4 | 24 | 66 | 71 | 80 | 6 |
| 19 | 20 | 2 | 2 | 3 | 4 | 25 | 43 | 50 | 59 | 0 |
| 20 | 17 | 2 | 2 | 2 | 4 | 17 | 56 | 61 | 78 | 9 |
| 21 | 20 | 1 | 2 | 1 | 4 | 25 | 67 | 79 | 90 | 7 |
| 22 | 19 | 2 | 2 | 3 | 4 | 10 | 36 | 44 | 40 | 0 |
| 23 | 20 | 2 | 3 | 1 | 5 | 22 | 53 | 59 | 68 | 5 |
| 24 | 20 | 1 | 1 | 2 | 3 | 22 | 53 | 66 | 71 | 4 |
| 25 | 19 | 2 | 2 | 1 | 4 | 22 | 53 | 63 | 75 | 9 |
| 26 | 25 | 1 | 2 | 3 | 4 | 6 | 56 | 73 | 63 | 0 |
| 27 | 17 | 2 | 3 | 2 | 4 | 27 | 66 | 67 | 76 | 7 |
| 28 | 18 | 1 | 2 | 1 | 4 | 15 | 59 | 75 | 92 | 12 |
| 29 | 20 | 2 | 2 | 2 | 4 | 15 | 42 | 44 | 61 | 6 |
| 30 | 39 | 2 | 2 | 3 | 4 | 3 | 36 | 43 | 44 | 0 |
| 31 | 18 | 2 | 3 | 3 | 4 | 21 | 50 | 67 | 56 | 0 |
| 32 | 25 | 1 | 1 | 2 | 4 | 6 | 35 | 31 | 54 | 6 |
| 33 | 19 | 2 | 2 | 1 | 3 | 16 | 57 | 59 | 77 | 6 |
| 34 | 18 | 2 | 1 | 1 | 4 | 21 | 51 | 74 | 76 | 8 |
| 35 | 18 | 2 | 2 | 3 | 5 | 9 | 45 | 66 | 50 | 0 |
| 36 | 18 | 2 | 2 | 2 | 4 | 16 | 40 | 60 | 61 | 7 |
| 37 | 18 | 1 | 1 | 3 | 3 | 17 | 40 | 52 | 63 | 0 |
| 38 | 19 | 2 | 1 | 2 | 5 | 25 | 70 | 79 | 91 | 5 |
| 39 | 17 | 2 | 2 | 1 | 4 | 22 | 76 | 86 | 84 | 10 |
| 40 | 22 | 1 | 1 | 1 | 4 | 24 | 71 | 94 | 98 | 5 |
| 41 | 19 | 2 | 3 | 2 | 4 | 22 | 78 | 78 | 99 | 6 |
| 42 | 19 | 2 | 2 | 3 | 4 | 23 | 44 | 70 | 74 | 0 |

*Note.* Sex 1 = male, 2 = female; Glasses 1 = yes, 2 = no, 3 = reading/writing only; Thought condition 1 = counterfactual, 2 = prefactual, 3 = control; Baseline motivation 1 = not at all motivation, 2 = not motivated, 3 = unsure, 4 = motivated, 5 = very motivated.
