# Supplementary material for: Improving Physical Task Performance with Counterfactual and Prefactual Thinking
Source: PLoS One. 2016 Dec 12;11(12):e0168181. doi: 10.1371/journal.pone.0168181 (PMC5152910; doi:10.1371/journal.pone.0168181)
Supplement: S3 Table — (DOCX) [file pone.0168181.s004.docx]

**S3 Table. Dataset Experiment 2.**

| **Participant** | **Age** | **Sex** | **Thought Condition** | **Baseline Motivation** | **Practice Best Attempt** | **Trial 1**  **Best Attempt** | **Trial 2**  **Best Attempt** | **Trial 3**  **Best Attempt** | **Thoughts Generated** |
| --- | --- | --- | --- | --- | --- | --- | --- | --- | --- |
| 1 | 33 | 2 | 1 | 4 | 10.00 | 10.63 | 7.21 | 13.29 | 10 |
| 2 | 23 | 1 | 3 | 4 | 6.25 | 7.58 | 7.13 | 7.76 | 0 |
| 3 | 22 | 1 | 2 | 4 | 3.91 | 4.13 | 4.18 | 4.86 | 8 |
| 4 | 19 | 2 | 1 | 4 | 4.63 | 5.56 | 3.05 | 6.43 | 7 |
| 5 | 32 | 2 | 1 | 5 | 5.16 | 3.51 | 6.20 | 7.55 | 8 |
| 6 | 19 | 1 | 3 | 4 | 6.96 | 7.06 | 7.43 | 7.16 | 0 |
| 7 | 18 | 2 | 2 | 4 | 5.11 | 5.90 | 11.68 | 23.76 | 12 |
| 8 | 19 | 2 | 3 | 4 | 3.46 | 4.21 | 10.03 | 10.55 | 0 |
| 9 | 23 | 2 | 1 | 4 | 3.80 | 4.03 | 5.23 | 6.07 | 2 |
| 10 | 19 | 2 | 1 | 4 | 2.45 | 2.51 | 3.38 | 3.60 | 8 |
| 11 | 21 | 2 | 1 | 4 | 4.93 | 5.13 | 8.93 | 9.60 | 6 |
| 12 | 18 | 1 | 2 | 4 | 4.05 | 3.88 | 6.11 | 6.46 | 9 |
| 13 | 24 | 2 | 3 | 4 | 9.33 | 11.08 | 11.08 | 21.48 | 0 |
| 14 | 24 | 1 | 1 | 4 | 5.65 | 9.21 | 25.55 | 36.36 | 9 |
| 15 | 21 | 2 | 2 | 4 | 6.91 | 16.21 | 22.66 | 36.41 | 7 |
| 16 | 22 | 2 | 1 | 4 | 25.71 | 27.18 | 30.52 | 40.60 | 5 |
| 17 | 31 | 1 | 2 | 4 | 5.16 | 5.48 | 12.25 | 16.11 | 5 |
| 18 | 18 | 1 | 2 | 1 | 4.18 | 13.81 | 8.20 | 17.85 | 6 |
| 19 | 22 | 2 | 3 | 4 | 6.25 | 13.76 | 38.75 | 36.46 | 0 |
| 20 | 25 | 2 | 2 | 4 | 5.35 | 4.31 | 5.81 | 6.83 | 7 |
| 21 | 21 | 2 | 3 | 2 | 3.43 | 3.40 | 4.08 | 3.58 | 0 |
| 22 | 19 | 2 | 3 | 4 | 9.50 | 5.05 | 6.66 | 7.01 | 0 |
| 23 | 23 | 2 | 1 | 4 | 12.19 | 17.21 | 22.90 | 28.43 | 5 |
| 24 | 34 | 2 | 2 | 4 | 7.60 | 9.73 | 20.73 | 27.48 | 8 |
| 25 | 19 | 2 | 1 | 4 | 13.82 | 11.90 | 17.86 | 20.90 | 5 |
| 26 | 22 | 2 | 2 | 4 | 4.10 | 6.53 | 7.75 | 5.31 | 9 |
| 27 | 22 | 2 | 2 | 4 | 7.11 | 9.81 | 12.06 | 11.60 | 7 |
| 28 | 23 | 2 | 2 | 4 | 2.81 | 4.27 | 5.71 | 12.14 | 6 |
| 29 | 40 | 2 | 3 | 4 | 4.96 | 4.08 | 5.98 | 4.56 | 0 |
| 30 | 20 | 2 | 3 | 4 | 4.28 | 22.88 | 14.35 | 24.65 | 0 |
| 31 | 24 | 1 | 1 | 4 | 5.26 | 6.16 | 6.56 | 13.28 | 8 |
| 32 | 22 | 1 | 3 | 4 | 3.45 | 6.30 | 10.43 | 7.26 | 0 |
| 33 | 21 | 2 | 2 | 5 | 28.71 | 14.01 | 13.80 | 19.33 | 5 |
| 34 | 21 | 1 | 3 | 5 | 5.58 | 6.33 | 18.33 | 13.19 | 0 |
| 35 | 26 | 1 | 1 | 4 | 34.06 | 42.40 | 48.91 | 69.16 | 8 |
| 36 | 20 | 1 | 1 | 4 | 8.96 | 14.91 | 16.04 | 32.18 | 5 |
| 37 | 26 | 2 | 3 | 4 | 3.38 | 4.93 | 4.25 | 5.06 | 0 |
| 38 | 18 | 2 | 3 | 4 | 3.88 | 4.58 | 6.78 | 7.01 | 0 |
| 39 | 25 | 2 | 2 | 5 | 40.45 | 62.19 | 63.30 | 87.08 | 8 |
| 40 | 25 | 2 | 1 | 4 | 3.98 | 3.86 | 3.35 | 4.48 | 6 |
| 41 | 20 | 2 | 2 | 4 | 4.11 | 3.31 | 5.68 | 9.16 | 9 |
| 42 | 21 | 2 | 3 | 4 | 3.26 | 2.90 | 6.01 | 4.58 | 0 |

*Note.* Sex 1 = male, 2 = female; Thought condition 1 = counterfactual, 2 = prefactual, 3 = control; Baseline motivation 1 = not at all motivation, 2 = not motivated, 3 = unsure, 4 = motivated, 5 = very motivated.
